# Assessment of risk factors associated with outpatient parenteral antimicrobial therapy (OPAT) complications: A retrospective cohort study

**DOI:** 10.1017/ash.2022.313

**Published:** 2022-11-11

**Authors:** Christina M. Kaul, Matthew Haller, Jenny Yang, Sadie Solomon, Yaojie Wang, Rong Wu, Yu Meng, Robert A. Pitts, Michael S. Phillips

**Affiliations:** 1 Division of Infectious Diseases, NYU Grossman School of Medicine, New York, New York; 2 NYU Grossman School of Medicine, New York, New York; 3 Department of Internal Medicine, NYU Grossman School of Medicine, New York, New York; 4 Department of Infection Control and Prevention, NYU Langone Health, New York, New York; 5 NYU School of Global Public Health, New York, New York

## Abstract

**Objective:**

To characterize factors associated with increased risk of outpatient parenteral antimicrobial therapy (OPAT) complication.

**Design:**

Retrospective cohort study.

**Setting:**

Four hospitals within NYU Langone Health (NYULH).

**Patients:**

All patients aged ≥18 years with OPAT episodes who were admitted to an acute-care facility at NYULH between January 1, 2017, and December 31, 2020, who had an infectious diseases consultation during admission.

**Results:**

Overall, 8.45% of OPAT patients suffered a vascular complication and 6.04% suffered an antimicrobial complication. Among these patients, 19.95% had a 30-day readmission and 3.35% had OPAT-related readmission. Also, 1.58% of patients developed a catheter-related bloodstream infection (CRBSI). After adjusting for key confounders, we found that patients discharged to a subacute rehabilitation center (SARC) were more likely to develop a CRBSI (odds ratio [OR], 4.75; *P* = .005) and to be readmitted for OPAT complications (OR, 2.89; *P* = .002). Loss to follow-up with the infectious diseases service was associated with increased risks of CRBSI (OR, 3.78; *P* = .007) and 30-day readmission (OR, 2.59; *P* < .001).

**Conclusions:**

Discharge to an SARC is strongly associated with increased risks of readmission for OPAT-related complications and CRBSI. Loss to follow-up with the infectious diseases service is strongly associated with increased risk of readmission and CRBSI. CRBSI prevention during SARC admission is a critically needed public health intervention. Further work must be done for patients undergoing OPAT to improve their follow-up retention with the infectious diseases service.

Outpatient parenteral antimicrobial therapy (OPAT) is used in the outpatient setting to treat certain infectious conditions that require a prolonged course of antimicrobials. However, an estimated 1 in 1,000 people in the United States receive OPAT annually, and the duration of therapy typically ranges from 2 to 6 weeks.^1,2^ Utilization of OPAT has increased over the years due to technological improvements as well as medical cultural changes including provider familiarity, cost-containment efforts, and patient preferences.^3–6^ OPAT is widely utilized for multiple conditions across a variety of populations and healthcare settings.^3,7–9^

Given that OPAT has transitioned to a standard of care, treatment outcomes and complications have been increasingly studied over the past decade. Complications are typically categorized in the following groups: vascular-access–related complications, antimicrobial-related complications, 30-day readmission, and death. Vascular complication rates range from 0.05% to 16%.^10–14^

Despite increased reporting of outcomes, only few studies have assessed risk factors for increased complications; additionally, these studies have not found consistent risk factors across demographics. In one study of vascular complications, some identified risk factors included longer OPAT duration, younger age, female sex, and intravenous drug use (IVDU).^11^ Another study identified longer line duration as a potential risk factor,^15^ and a third study revealed the use of midline catheters, the presence of *Staphylococcus aureus* infections, and the use of vancomycin or daptomycin as potential causes for increased risk.^16^ In studies of antimicrobial complications, use of vancomycin and ertapenem have been identified as possible risk factors.^10,12^ Some risk factors that have been identified for 30-day readmission include homecare OPAT, aged >70 years,^17^ discharge to long-term care facilities (LTCFs), or discharge to a subacute rehabilitation center (SARC).^18,19^ Given the wide range of reported OPAT complications and lack of reproduced results, further work is required to solidify our understanding.

We conducted an analysis of all patients receiving OPAT after discharge from 4 hospitals across a single health system over a 4-year period. We have described the clinical factors associated with an increased risk of OPAT complications, including vascular-access–related complications, antimicrobial-related complications, and 30-day readmission. Although prior study of risk factors in OPAT outcomes has focused on 1 complication type, we assessed multiple outcomes. Our research builds upon the limited body of literature that has assessed differential complication rates in the OPAT population to identify populations most at risk.

## Methods

### Study design and setting

This retrospective cohort study was conducted at 4 hospitals within the NYU Langone Health (NYULH) system in the New York City region, representing >1,800 total beds, between January 2017 and December 2020, including NYU Tisch (site a), NYU Winthrop (site b), NYU Brooklyn (site c), and NYU Langone Orthopedic Hospital (site d). The study was approved and granted a waiver of authorization and informed consent by the NYU Institutional Review Board.

Prior to discharge, the OPAT process at NYULH consists of 4 steps: (1) The patient satisfies criteria for OPAT via an inpatient evaluation by the infectious diseases team. (2) The patient agrees to OPAT after shared decision making discussions with the inpatient primary team. (3) The inpatient team informs the outpatient OPAT team about impending discharge. And (4) a dedicated OPAT outpatient service evaluates the patient, provides essential education, and is responsible for longitudinal follow-up until the antimicrobial course is completed. Patients at sites a and d were managed by the same outpatient OPAT team, and sites b and c were managed by their respective OPAT teams. Antimicrobial regimens were recommended by the inpatient infectious diseases team and were reviewed by the outpatient OPAT team. Patients were recommended to be scheduled for outpatient follow-up with an infectious disease physician within 2–4 weeks of discharge, and telephone visits with the nurse practitioner were scheduled at regular intervals, typically weekly or more frequently as needed. In the event of unattended appointments, clinic staff and nurse practitioners would make attempts to reach patients, family members, or facility staff at discharge locations by telephone. At the completion of OPAT, vascular access was removed in the infectious disease clinic or by facility staff after verbal confirmation that was read back by the nurse practitioner.

### Case ascertainment and inclusion and exclusion criteria

All OPAT episodes in patients aged ≥18 years initiated between January 1, 2017, and December 31, 2020, were included in this study. An episode of OPAT for inclusion was defined as the administration of at least 2 doses on different days without intervening hospitalization according to the guidelines in the Infectious Diseases Society of America clinical practice guideline.^6^ The study included patients aged ≥18 years, admitted to an acute-care facility at NYULH, who had an infectious diseases consultation during admission. Other indications for outpatient vascular access included chemotherapy, intravenous hydration, and other noninfectious indications that were not relevant to this study.

### Data sources and data extraction

Sources of data included the electronic health record (EHR) with Epic software (Epic Systems, Verona, WI) and the separate, hospital-based, NYULH OPAT registry. Records of all patients who were discharged with a peripherally inserted central catheter (PICC) or midline catheter were extracted from the EHR, and all records of cases of OPAT were manually screened by investigators for inclusion in the study. All patients who are discharged on OPAT with follow-up at NYULH are tracked in the NYULH OPAT registry, which was used to confirm the accuracy of the data extraction. Data were extracted by a member of the study team. After receiving the data, study personnel approved by our institutional review board conducted a comprehensive chart review of 1,846 OPAT episodes. Reviewers collected detailed data regarding complications and OPAT potential risk factors directly from patient records using a defined protocol built from the following definitions.

### Definitions

OPAT complications were divided into 3 categories: vascular-access–related, antimicrobial-related, and 30-day readmission. Vascular-access–related complications included vascular catheter occlusion, vascular catheter thrombosis, vascular catheter dislodgement, and central-line–associated bloodstream infection.^6,10–12,17^ Antimicrobial-related complications included laboratory issues, adverse drug reaction, *Clostridioides difficile* infection. Laboratory issues included acute kidney injury (AKI), hyperkalemia, cytopenia, elevated liver enzymes, elevated creatine kinase, and eosinophilia.^6,10,12,17^ Drug reactions included any response noticed by the patient that were noxious or unintended, such as rash. All-cause 30-day readmission and OPAT-related readmissions were recorded. The definition of vascular-access complications was adapted from Shreshtha et al^16^ and was defined as a vascular access complication that triggered an intervention. A CRBSI was defined according to the Centers for Disease Control and Prevention National Healthcare Safety Network case definition.^20^ Vascular catheter occlusion was defined as patient or caregiver inability to infuse the intravenous antimicrobial because of lack of flow. Venous thrombosis was defined as a finding of deep or superficial venous thrombosis in the blood vessel with the vascular device or proximal to it. All-cause 30-day readmission was defined as a patient being readmitted within 30 days of discharge for any reason. An OPAT-related admission was defined as a readmission due to a recorded OPAT complication as previously described.

Potential risk factors in 4 domains were chosen for study based on previously published data: demographics, health-related factors, vascular-access characteristics, and OPAT-treatment–related factors. Demographic factors included sex, race, employment status, and language discordance. Language discordance was defined with the dyad of patient and infectious diseases provider because the provider recommended and provide initial counseling on OPAT. Location of discharge included home, subacute rehabilitation center (SARC), acute rehabilitation center (ARC), and LTCF. Patient discharge locations were determined by the primary medical or surgical team in collaboration with the case management team and the patient. Health-related factors included the presence of relevant medical comorbidities including diabetes mellitus, chronic kidney disease, liver disease, malignancy, and HIV status. Current and history of IVDU were also recorded. Vascular-access characteristics included the type of venous access placed, duration of venous access, and the number of times the venous catheter was accessed per day for antimicrobial administration. OPAT-treatment–related factors included loss to follow-up with the infectious diseases service and discharge location. Loss to follow-up with the infectious diseases service was defined as patients who did not have any contact with an infectious disease physician or nurse practitioner after hospital discharge during the OPAT course.

Race data were self-reported by patients, and race categories were defined by the investigators based on the US Office of Management and Budget Revisions to the Standards for the Classification of Federal Data on Race and Ethnicity as follows: Asian, Non-Hispanic Black (hereafter, Black), Hispanic or Latino, and non-Hispanic White (hereafter, White). We excluded individuals who self-reported being of other race and ethnicity and those who were of unknown race and ethnicity because of the small sample size.

### Statistical analysis

Data were analyzed using SPSS version 28 software (IBM, Armonk, NY) and Microsoft Office Excel 2019 (Microsoft, Redmond, WA). Prior to hypothesis testing, missing data patterns and descriptive analyses for each study variable were examined to evaluate data quality. Descriptive statistics were used to describe the study population. Patients without any encounters noted in the EHR after discharge were considered completely lost to follow-up and were excluded from outcomes analysis but were included in demographic descriptive statistics. A multivariate logistic regression model was used to examine factors associated with OPAT complication risk, adjusted for key covariates including chronic medical condition, hospital of admission, age, line duration, antimicrobial dosing frequency, and line type. Antimicrobial regimen was utilized in the regression model analyzing antimicrobial-related complications. Chronic medical condition was treated as a dichotomous categorical variable. *P* ≤ .05 was considered statistically significant.

## Results

**Fig. 1.**
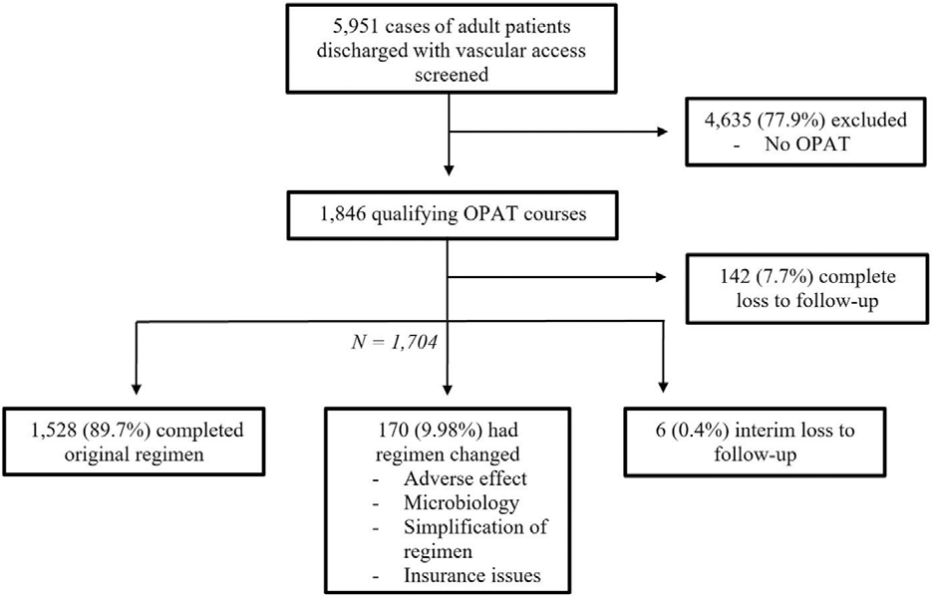
Study enrollment flow diagram.

Between January 1, 2017, and December 31, 2020, 5,951 cases of adult patients discharged with vascular access were identified in the EHR. After manual review, 22.1% were determined to be OPAT courses that fit our inclusion criteria. Of these courses, sufficient follow-up data were available for 92.3%, but 7.7% were completely lost to follow-up after hospital discharge. Antimicrobial regimens were switched due to adverse effects, simplification of regimen, underinsurance, or microbiological indication (Fig. 1). Changes in antimicrobial regimen were similar across sites a, c, and d (9.1%, 6.2%, 8.2%, respectively), with a higher rate noted at site b (23.5%).

In total, 779 patients (42.2%) in the study cohort were female. In addition, 1,042 (60.8%), were White, 294 (17.2%) were Hispanic, 234 (13.7%) were Black, and 144 (8.4%) were Asian. Moreover, 1,036 patients (56.1%) had midline catheters and 810 (43.9%) had PICCs. The insurance of 469 patients (25.4%) was commercial, and the remaining patients (74.6%) had federal insurance; no patients were uninsured.

Most patients (59.2%) were discharged to receive OPAT at home. A large proportion of patients (30.5%) were discharged to an SARC. Also, 43.1% of patients were treated for noninvasive conditions (eg, pneumonia, urinary tract infection, or cellulitis). Carbapenems were noted to be the most used antimicrobial (26.8%), followed closely by cephalosporins (26.7%). The complete baseline characteristics of the study population are provided in Table 1.

**Table 1.** Patient Characteristics

Characteristic	No. (%) or Median (IQR)
Age, y	66 (55–76)
Sex, female	779 (42.2)
**Race and ethnicity**	
Non-Hispanic White	1,042 (60.8)
Hispanic	294 (17.2)
Black	234 (13.7)
Asian	144 (8.4)
Intravenous drug use	45 (2.4)
**Insurance type**	
Private	469 (25.4)
Federal	1,377 (74.6)
**Line type**	
Peripherally inserted central catheter	810 (43.9)
Midline catheter	1,036 (56.1)
Line duration	22 (11–39)
Antimicrobial duration	23 (13–41)
Dosing frequency (per day)	2 (1–3)
**Discharge location**	
Home	1,093 (59.2)
SARC	563 (30.5)
ARC	125 (6.8)
LTCF	65 (3.5)
**OPAT indication**	
Noninvasive (eg, pneumonia, UTI, cellulitis)	796 (43.1)
Deep abscess	300 (16.3)
Osteomyelitis	299 (16.2)
Prosthetic joint infection	145 (7.9)
Endovascular infection or infective endocarditis	100 (5.4)
Non–joint device or hardware infection	41 (2.2)
Septic arthritis	38 (2.1)
Other	127 (6.9)
**Antimicrobial category**	
Carbapenem	494 (26.8)
Cephalosporin	493 (26.7)
Penicillin	271 (14.7)
Combination therapy	247 (13.4)
Vancomycin	176 (9.5)
Daptomycin	56 (3.0)
Antifungal	25 (1.4)
Antiviral	20 (1.1)
Monobactam	13 (0.7)
Other	25 (1.4)

Note. IQR, interquartile range; OPAT, outpatient parenteral antimicrobial therapy; UTI, urinary tract infection; SARC, subacute rehabilitation center; ARC, acute rehabilitation center; LTCF, long-term care facility.

Moreover, 8.45% of courses resulted in vascular complications: 90 (62.5%) of complications occurred in patients with midline catheters and 54 (37.5%) occurred in patients with PICCs. Line dislodgement or leak was the most common complication, being present in 4.40% of all OPAT courses. Also, 1.46% of all courses were complicated by a CRBSI, correlating with a rate of 0.57 infections per 1,000 central-line days. Of the 144 cases with vascular-access complications, 33 (22.9%) resulted in readmission, whereas 6.04% of courses resulted in antimicrobial-related complications. The choice of antimicrobial regimen was not independently associated with antimicrobial-related complication risk. Furthermore, 20% of courses resulted in 30-day readmission, 57 (3.35%) of which were a direct result of OPAT-associated complications. In this study, no deaths were related to OPAT administration. A complete description of complication results is provided in Table 2.

**Table 2.** OPAT Complications

Complication Type	OPAT Courses, No. (%)	Event Rate per 1,000 Days
**Vascular**	144 (8.45)	2.80
CRBSI	27 (1.58)	0.57
Dislodgment, leak, or bleeding	75 (4.40)	1.44
Occlusion	27 (1.58)	0.53
Thrombosis	10 (0.59)	0.19
Line-adjacent cellulitis or rash	8 (0.47)	0.18
**Antimicrobial**	103 (6.04)	2.01
*Clostridioides difficile* infection	7 (0.41)	0.14
**30**	340 (19.95)	
OPAT	57 (3.35)	

Note. OPAT, outpatient parenteral antimicrobial therapy; CRBSI, catheter-related bloodstream infection.

**Table 3.** Patient Risk Factors in Vascular Access-Related Complications

Variable	Vascular Complications			CRBSI		
	OR	95% CI	*P* Value	OR	95% CI	*P* Value
Sex, female	1.405	[0.95–2.07]	.089	1.372	[0.57–3.31]	.482
Ethnicity						
Asian	0.700	[0.29–1.70]	.431	2.442	[0.44–13.2]	.302
Black	0.814	[0.44–1.50]	.506	0.921	[0.024–3.70]	.906
Hispanic	0.867	[0.50–1.52]	.617	1.718	[0.50–6.13]	.400
Language discordance	0.901	[0.51–1.59]	.720	0.635	[0.16–2.51]	.516
IVDU	3.287	[0.45–1.31]	.012	4.312	[0.69–25.5]	.113
Employed	1.020	0.57–1.77]	.942	0.799	[0.24–2.86]	.720
Loss to follow-up with ID service	0.766	[0.45–1.31]	.328	3.781	[1.47–10.32]	**.007**
Discharge location						
SARC	0.989	[0.60–1.61]	.966	4.745	[1.62–14.2]	**.005**
ARC	0.682	[0.29–1.64]	.392	2.284	[0.44–12.9]	.340
LTCF	1.064	[0.30–3.73]	.923	0.180	[0.57–22.2]	.180

Note. CRBSI, catheter-related bloodstream infection; OR, odds ratio; CI, confidence interval; ID, infectious diseases; IVDU, intravenous drug use; SARC, subacute rehabilitation center; ARC, acute rehabilitation center; LTCF, long-term care facility. Bold indicates statistical significance.

**Table 4.** Patient Risk Factors in Antimicrobial-Related Complications

Variable	OR	95% CI	*P* Value
Sex, female	0.875	[0.54–1.39]	.575
**Ethnicity**			
Asian	0.686	[0.26–1.81]	.446
Black	1.376	[0.72–2.70]	.344
Hispanic	1.084	[0.57–2.10]	.809
Language Discordance	0.897	[0.46–1.72]	.746
Employed	1.272	[0.66–2.58]	.484
**Discharge location**			
SARC	0.777	[0.44–1.37]	.777
ARC	0.078	[0.01–0.67]	.018
LTCF	0.375	[0.05–2.90]	.375

Note. OR, odds ratio; CI, confidence interval; SARC, subacute rehabilitation center; ARC, acute rehabilitation center; LTCF, long-term care facility.

**Table 5.** Patient Risk Factors in 30-Day Readmission

Variable	30-Day Readmission			OPAT-Related Readmission		
	OR	95% CI	*P* Value	OR	95% CI	*P* Value
Sex, female	1.125	[0.84–1.51]	.430	1.613	[0.91–2.85]	.100
**Ethnicity**						
Asian	0.815	[0.44–1.51]	.517	0.720	[0.20–2.61]	.618
Black	0.861	[0.54–1.36]	.524	0.830	[0.35–1.98]	.675
Hispanic	1.086	[0.72–1.63]	.690	0.778	[0.32–1.89]	.579
Language discordance	1.049	[0.70–1.57]	.818	1.128	[0.50–2.55]	.771
IVDU	2.170	N/A	.061	0.592	[0.07–4.88]	.626
Employed	0.942	[0.62–1.43]	.778	1.154	[0.50–2.64]	.734
Loss to Follow-Up with IDservice	2.586	[1.85–3.62]	**<.001**	1.847	[0.95–3.57]	.069
Discharge location						
SARC	1.025	[0.73–1.44]	.890	2.885	[1.49–5.58]	**.002**
ARC	0.417	[0.21–0.84]	**.014**	0.727	[0.16–3.20]	.674
LTCF	1.045	[0.47–2.34]	.914	2.356	[0.61–9.08]	.213

Note. OR, odds ratio; CI, confidence interval; ID, infectious diseases; SARC, subacute rehabilitation center; ARC, acute rehabilitation center; LTCF, long-term care facility. Bold indicates statistical significance.

The multivariate logistic regression models were adjusted for age, presence of chronic medical conditions, hospital of admission, line duration, line type, and dosing frequency. Regarding vascular-access–related complications, a history of IVDU (OR, 3.29; 95% CI, 1.30–8.29; *P* = .012) was associated with increased risk. Similar to previous literature, and midline catheter use (compared to PICC use) was associated with an increased risk of vascular-access–related complications. Discharge to an SARC (OR, 4.74; 95% CI, 1.60–14.03; *P* = .005) and loss to follow-up with outpatient infectious diseases staff (OR, 3.78; 95% CI, 2.81–4.75; *P* = .007) were associated with increased risk of CRBSI. Line duration and daily dosing frequency were not independently associated with increased vascular-access–related complications. We did not identify disparities in vascular-access–related complication risk based on sex, race or ethnicity, language discordance, or employment status (Table 3). Regarding antimicrobial-related complications, discharge to an ARC was associated with a decreased risk (OR, 0.078; 95% CI, −2.03 to 2.89; *P* = .018). Antimicrobial-related complications were associated with antimicrobial regimen. We did not identify disparities in antimicrobial-related complication risk based on sex, race or ethnicity, language discordance, or employment status (Table 4). Loss to follow-up with the outpatient infectious diseases service was associated with an increased risk of 30-day readmission (OR, 2.59; 95% CI, 2.25–2.92; *P* < .001). Discharge to an ARC was associated with a decreased risk of 30-day readmission (OR, 0.42; 95% CI, 0.28–1.12; *P* = .014). Discharge to an SARC was associated with an increased risk of OPAT-related 30-day readmission (OR, 2.89; 95% CI, 1.49–5.59; *P* = .002). We did not identify disparities in 30-day readmission rate based on sex, race or ethnicity, language discordance, or employment status (Table 5).

Our findings highlight the differential risk of OPAT complications across multiple patient outcomes. OPAT has emerged as a mainstay of practice, decreasing the need for inpatient hospitalization for long-term antimicrobial administration. However, challenges are still present in its delivery, leading to complications in the outpatient setting. In our study, a slight majority of patients were discharged home for therapy. However, a large proportion of patients were discharged to skilled nursing facilities specifically for OPAT, reflecting challenges in administration at home without continued support from healthcare providers, as well as the importance of including these facilities when targeting interventions to improve outcomes.

Although many studies have described the population receiving OPAT, few studies have assessed which populations are most at risk for complications. In our study, the use of midline catheters was associated with an increase in vascular-access–related complications, which is in line with a prior study.^16^ Dislodgements were common in midline catheters, which is not surprising given the shorter length of the tubing. Patients with reported IVDU were also noted to have increased vascular complications, the vast majority of which were line dislodgements. The reasons for this finding were not elucidated by our research. Notably, several previous studies have reported that OPAT completion rates are similar in patients with reported IVDU compared to those without, and vascular-access–related complications have been reported as similar in both groups.^21–24^

Discharge to an ARC was associated with decreased risk of antimicrobial complications, as well as a decreased risk of 30-day readmission. These conclusions are not surprising given the robust healthcare services provided in this continued acute-care environment.

Although not defined as an outcome variable in this study, loss to follow-up with the outpatient infectious diseases service was associated with increased risk of CRBSI and 30-day readmission. This difference remained even when limiting the analysis to patients receiving therapy for >14 days or when excluding noninvasive indications for OPAT. The reasons for these specific differences remain unclear and warrant further attention.

A most interesting conclusion was the association between discharge to an SARC and increased risk for CRBSI- and OPAT-related readmission, which remained after adjusting for key covariates. This difference remained in a limited analysis of patients receiving therapy for >14 days and when excluding noninvasive indications for OPAT. Prior studies have identified discharge to an SARC as a risk factor for increased readmission, which is also supported by our work,^18,19^ but no prior studies have identified an association between admission to an SARC and CRBSI. The reason for this association is not clear but should certainly be incorporated into future interventions created to improve patient outcomes in OPAT.

The strengths of our study include the large sample size, long-term data over multiple years and across multiple hospitals, a racially and ethnically diverse patient population, and a diversity of antimicrobials used. Our study also had limitations. Although 4 sites were included, the study was conducted at a single health system, which may limit generalizability. Despite a racial and ethnic distribution reflects the population in the United States, all patients in the study had health insurance, which could have affected results. The understudied issue of access to OPAT is of interest and could be important for future study. Additionally, 7.7% of patients were completely lost to follow-up, which could have affected our results.

After adjusting for key confounders, we found that discharge to an SARC was strongly associated with increased risk of readmission for OPAT-related complications and CRBSI, Solidifying an understanding of differences in OPAT complications in the broader population would allow for targeted education efforts and improved provider understanding of vulnerable patients. Interventions that target patients discharged to an SARC should be considered and utilized when creating programs to support patients discharged with OPAT. Further work is required to optimize therapeutic strategies for safe OPAT delivery across diverse populations.
